# Frequency and factors associated with the preference for self-testing for human papillomavirus detection

**DOI:** 10.17843/rpmesp.2025.422.14372

**Published:** 2025-06-19

**Authors:** María A. Rosas-Mendoza, Yuly R. Santos-Rosales, Marco A. Chilipio-Chiclla

**Affiliations:** 1 Universidad Nacional Mayor de San Marcos, Faculty of Medicine, Lima, Peru. Universidad Nacional Mayor de San Marcos Universidad Nacional Mayor de San Marcos Faculty of Medicine Lima Peru; 2 San Juan de Lurigancho Hospital, Lima, Peru. San Juan de Lurigancho Hospital Lima Peru

**Keywords:** Human Papillomavirus DNA Tests, Human Papillomavirus Viruses, Mass Screening, Self-administration

## Abstract

**Objective.:**

To analyze the frequency and factors associated with self-sampling preference for detecting human papillomavirus (HPV) in Peruvian women at a healthcare center, 2023-2024.

**Materials and methods.:**

A cross-sectional analytical study was conducted. The sample consisted of 275 women aged 30 to 49 years who from the Rinconada Healthcare Center. The main variable was evaluated directly using a dichotomous question, which revealed the user’s preference for this method of cervical sample collection for HPV detection. The instrument was a valid and reliable questionnaire. Multivariate statistics were used to find the associated factors.

**Results.:**

We found that 75.6% of users prefer self-sampling for HPV detection. Adjusted analysis showed that higher education (RPa=1.28; 95% CI: 1.01-1.64) and secondary education (RPa=1.46; 95% CI: 1.11-1.92) increased the likelihood of preferring self-sampling; while cohabiting marital status (RPa=0.61; 95% CI: 0.44-0.83), being from the rest of the coast (RPa=0.70; 95% CI: 0.50-0.97), Catholic religion (RPa=0.84; 95% CI: 0.74-0.96) and having a higher quartile of knowledge about HPV (RPa=0.88; 95% CI: 0.71-0.95) reduced the likelihood of preferring self-sampling.

**Conclusion.:**

The frequency of preference for self-sampling for HPV detection is high and is associated with secondary/higher education, cohabitation, living on the coast except for the capital, being Catholic, and having a higher level of knowledge about HPV.

## INTRODUCTION

Cervical cancer, caused by the human papillomavirus (HPV), is a global public health problem with high mortality rates among women, especially in low-income countries ^1^. In 2022, the Global Cancer Observatory (GLOBOCAN) recorded 4,809 new cases of cervical cancer and 2,545 deaths attributed to this cause in Peru ^2^.

This type of cervical cancer is easily preventable, and public health interventions can be effective in addressing this problem. Such interventions include screening as a preventive measure. Different types of screening are used in Peru, such as cervical cytology or PAP smears, visual inspection with acetic acid (VIA), and molecular tests (self-sampling) for HPV detection; the latter can be collected by the woman herself ^3^. PAP and VIA have several obstacles that limit their effectiveness, including problems with sample quality, as well as sociocultural and psychological barriers ^4^^,^^5^. Self-sampling has been shown to be an acceptable, preferred, and effective alternative, especially in vulnerable populations ^6^^,^^7^, understood as the self-administered procedure that allows the user to collect the cervicovaginal sample using specific devices, following standardized instructions and without the direct intervention of a healthcare professional ^8^.

The World Health Organization (WHO) recommends self-sampling as a primary screening test because it is more sensitive than conventional cytology ^1^^)^ and has proven to be highly accepted and preferred in different populations in Asia, Africa, and Latin America. In Mexico, screening coverage increased in marginalized populations, and this experience was replicated in Argentina. In addition, countries such as Chile and Colombia have implemented self-sampling as part of screening for cervical cancer prevention ^1^^,^^6^^,^^7^.

Self-sampling reduces existing gaps in access to cervical cancer screening tests, thereby increasing the coverage of screened women ^9^. It is crucial to assess preferences for self-sampling, as this provides a broader picture of how to intervene effectively in the population. The availability of a service does not guarantee its acceptance or preference by the target population. Therefore, understanding this aspect will facilitate the adaptation of public health strategies and prevention programs to meet community needs. This, in turn, will promote greater participation, contributing significantly to the prevention and control of cervical cancer.

The *La Rinconada* health center is a level I-2 primary healthcare center that provides preventive and promotional services, including cervical screening. As part of the cervical cancer prevention strategies promoted by the Ministry of Health (MINSA), HPV screening assisted by health professionals is being implemented. However, in order to expand coverage and facilitate access to more women, it is essential to understand their preferences regarding the method of sample collection and associated factors. Therefore, this study aimed to analyze the frequency and factors associated with the preference for self-sampling to detect human papillomavirus in Peruvian women at a healthcare center between February 2023 and February 2024.

**KEY MESSAGES**

Motivation for the study. Cervical screening is key to early detection of cancer, but its acceptability limits its implementation. It is essential to understand preferences for self-sampling to facilitate its implementation.

Findings. Factors such as educational level, marital status, place of origin, religion, and knowledge are associated with a preference for self-sampling to detect human papillomavirus.

Implications. Health authorities can use these findings to strengthen the promotion of self-sampling through educational campaigns focused on populations with factors that limit its acceptance, thereby improving screening coverage.

## MATERIALS AND METHODS

### Design and type of research

Observational, analytical, and cross-sectional study.

### Population and sample

The study was conducted at the *La Rinconada* Healthcare Center, located in San Juan de Miraflores (Lima, Peru). This facility was selected because of its immediate accessibility to the principal investigator. It is a level I-2 healthcare center that receives a predominantly low socioeconomic population. Data collection took place between December 2023 and February 2024.

The population comprised 951 women aged 30 to 49, this data was obtained from the register of the population assigned to the healthcare center. Women who agreed to participate voluntarily were included. Women with disabilities were excluded; however, during data collection, none presented physical, motor, or intellectual disabilities that would prevent their participation in the study.

The Netquest software (https://www.netquest.com/es/panel/calculadora-muestras/calculadoras-estadisticas) was used to calculate the sample size, considering a confidence level of 95% and a 5% margin of error. To ensure a robust and conservative statistical design, we assumed a 50% probability of success, which maximizes the variance of the expected proportion and ensures the largest sample size necessary to achieve the established confidence level and accuracy. Thus, a sample of 275 women was estimated, selected non-probabilistically for convenience.

### Variables and instrument

The main variable was the sampling method used for HPV detection, assessed directly through the question: “In your next cervical cancer screening, which would you prefer?” Two response options were offered: i) “self-sampling,” reflecting the user’s preference for this method of cervical sample collection, and ii) “test performed by a health professional,” indicating a preference for sampling assisted by a trained professional. Furthermore, this method of measuring preference for self-sampling is consistent with the methodology used in previous studies ^10^^,^^11^.

Sociodemographic factors were included, such as age (30-39/40-49 years), level of education (no education-primary/secondary/higher), marital status (single-widowed/married/cohabiting), place of origin (metropolitan Lima/rest of the coast/jungle/abroad), religion (Catholic/non-Catholic), proximity to healthcare center (1-10/11-30/31 minutes or more), and employment status (full-time work/part-time work/housewife).

Behavioral factors such as age at first sexual intercourse (≤17/>17 years), number of sexual partners (quantity), use of contraception (yes/no), number of children (quantity), last PAP smear (less than two years/more than two years/never), and history of self-sampling (yes/no).

Cognitive factors such as knowledge about human papillomavirus (≥75th percentile/<75th percentile) were assessed using a 16-item dichotomous (yes/no) questionnaire that addressed aspects related to HPV transmission, manifestations, epidemiology, and management, which was adapted from the study by Marlow *et al*. ^12^. One point was assigned for each correct answer, and a cutoff point was set at the 75th percentile to identify women in the highest knowledge quartile.

The instrument was a questionnaire (supplementary material 1) validated by five experts. Two of them were professional obstetricians with methodological experience, and three had extensive knowledge in gynecological cancer prevention. Content validity was assessed using Aiken’s V coefficient, obtaining a value of V=0.995, which indicates high validity. To determine reliability, Cronbach’s alpha coefficient was used. To this end, a pilot test was conducted on 30 women of childbearing age from the same healthcare center who were not included in the final sample. The coefficient was calculated using only polytomous response items. Results showed that the questionnaire has high reliability (0.72).

### Procedures

After obtaining the necessary permissions, the surveys were administered at the *La Rinconada* Healthcare Center. During the 75 working days of the study period (December 2023 to February 2024), four users were selected daily from the scheduled appointments. Only those who agreed to participate and signed the informed consent form were included. The surveys were self-administered in printed format and were conducted in the waiting room of the obstetrics area, being completed by the users themselves individually.

### Analysis plan

Data were stored in an Excel database, and all records of participants with missing information or inconsistencies were deleted. For data processing we used the Statistical Package for the Social Sciences (SPSS) version 26. For the descriptive analysis of the quantitative variable age, the median was used as a measure of central tendency and the interquartile range (IQR) as a measure of dispersion, after verifying the non-normality of the data using the Kolmogorov-Smirnov test. We used absolute frequencies (n) and relative frequencies (%) for the descriptive analysis of qualitative variables. We used Pearson’s chi-square test with a confidence level of 95% during the bivariate analysis. Finally, crude prevalence ratios (cPR) and adjusted prevalence ratios (aPR) were estimated using Poisson regression with robust variance, using the Generalized Linear Models interface. In the adjusted model, only variables with a p-value <0.20 in the crude model were included, following the criteria of Hosmer and Lemeshow ^13^, to reduce the risk of omitting relevant variables and to consider possible confounding factors.

### Ethical considerations

The Helsinki criteria and bioethical principles for human research were respected. The project was reviewed and approved by the Ethics Committee of the Faculty of Medicine of the Universidad Nacional Mayor de San Marcos (Document N°0152-2023) and the Ethics Committee of the Integrated Health Networks Directorate (DIRIS) Lima Sur (N° 047-2023).

## RESULTS

The general characteristics of the 275 women showed a median age of 37.0 years (IQR: 33.0-43.0). Most belonged to the 30-39 age group (62.2%), had a higher level of education (60.4%), and were married (66.9%). Most came from metropolitan Lima (56.0%), followed by those of foreign origin (27.3%). In addition, most were Catholic (65.8%), lived 31 minutes or more from the healthcare center (48.7%), and were housewives (71.3%) (Table 1).

**Table 1:** General characteristics of women aged 30 to 49 years old from the obstetrics area of the *La Rinconada* Healthcare center.

General characteristics		n	%
Age ^a^		37.0 (33.0-43.0)	
	30-39 years	171	62.2
	40-49 years	104	37.8
Education level			
	Higher education	166	60.4
	Secondary school	63	22.9
	Primary school	3	1.1
	No education	43	15.6
Marital status			
	Married	184	66.9
	Single	44	16.0
	Cohabitant	39	14.2
	Widow	8	2.9
Place of origin			
	Metropolitan Lima	154	56.0
	Rest of the coast	28	10.2
	Jungle	18	6.5
	Abroad	75	27.3
Religion			
	Catholic	181	65.8
	Evangelist	15	5.5
	Jehovah’s Witness	3	1.1
	Other ^b^	76	27.6
Proximity to healthcare center			
	31 min or more	134	48.7
	11 to 30 min	122	44.4
	1 to 10 min	19	6.9
Employment status			
	Full-time job	29	10.5
	Part-time job	50	18.2
	Housewife	196	71.3
Total		275	100.0

a Median and interquartile range, quantitative variable without normal distribution (Kolmogorov Smirnov test p<0.001).

b Includes those who stated that they did not practice any religion.

We found that 75.6% (208) of the women reported a preference for self-sampling for HPV detection, while 24.4% (67) showed a preference for sampling assisted by a healthcare professional.

The bivariate analysis revealed significant differences between women who preferred self-sampling and those who did not, in terms of educational level (p=0.010), marital status (p=0.001), place of origin (p=0.036), religion (p=0.008), and level of knowledge about HPV (p=0.019). Women who preferred self-sampling generally had a higher level of education (higher: 62.0%; secondary: 25.0%). In contrast, those who showed a preference for self-sampling were less likely to be cohabiting (9.6%), Catholic (61.5%), and have knowledge about HPV ≥ 75th percentile (44.7%). Other variables such as age, proximity to the healthcare center, employment status, age at first sexual intercourse, number of sexual partners, use of contraceptive methods, number of children, history of previous PAP tests, and history of HPV self-sampling did not show significant differences (p>0.05) according to preference for self-sampling (Table 2).

**Table 2:** Bivariate analysis of sociodemographic, behavioral, and knowledge characteristics according to self-sampling preference for HPV detection in women aged 30 to 49 years at the *La Rinconada* Health Center.

Characteristics		Does prefer self-sampling	Does not prefer self-sampling	p-value^a^
		n (%)	n (%)	
Age				
	40-49 years	73 (35.1)	31 (46.3)	0.101
	30-39 years	135 (64.9)	36 (53.7)	
Education level				
	Higher education	129 (62.0)	37 (55.2)	0.010
	Secondary school	52 (25.0)	11 (16.4)	
	No education/primary school	27 (13.0)	19 (28.4)	
Marital status				
	Cohabitant	20 (9.6)	19 (28.4)	0.001
	Married	146 (70.2)	38 (56.7)	
	Single/widow	42 (20.2)	10 (14.9)	
Place of origin				
	Rest of the coast	15 (7.2)	13 (19.3)	0.036
	Jungle	14 (6.7)	4 (6.0)	
	Abroad	57 (27.4)	18 (26.9)	
	Metropolitan Lima	122 (58.7)	32 (47.8)	
Religion				
	Catholic	128 (61.5)	53 (79.1)	0.008
	Not Catholic^b^	80 (38.5)	14 (20.9)	
Proximity to healthcare center				
	31 min or more	97 (46.6)	37 (55.2)	0.407
	11 to 30 min	97 (46.6)	25 (37.3)	
	1 to 10 min	14 (6.8)	5 (7.5)	
Employment status				
	Housewife	148 (71.2)	48 (71.6)	0.939
	Paid work	60 (28.8)	19 (28.4)	
Age of first sexual intercourse				
	>17 years	130 (62.5)	43 (64.2)	0.805
	< 17 years	78 (37.5)	24 (35.8)	
Number of sexual partners				
	2 +	131 (63.0)	42 (62.7)	0.965
	0-1	77 (37.0)	25 (37.3)	
Use of contraceptive methods				
	Yes	95 (45.7)	36 (53.7)	0.251
	No	113 (54.3)	31 (46.3)	
Number of children				
	3	91 (43.7)	31 (46.3)	0.702
	1 to 2	106 (51.0)	31 (46.3)	
	None	11 (5.3)	5 (7.4)	
PAP at any time				
	Yes	83 (39.9)	28 (41.8)	0.784
	No	125 (60.1)	39 (58.2)	
History of self-sampling to detect HPV				
	Yes	27 (13.0)	9 (13.4)	0.924
	No	181 (87.0)	58 (86.6)	
Knowledge about HPV				
	Percentile>75	93 (44.7)	41 (61.2)	0.019
	Percentile<75	115 (53.3)	26 (38.8)	
Total		208 (100.0)	67 (100.0)	

a Pearson Chi-square test.

b Includes those who claim not to profess any religion

PAP: Pap smear, HPV: Human papillomavirus

Crude analysis showed that the sociodemographic factors associated with the preference for self-sampling were higher education (PR=1.32; 95% CI: 1.02-1.71) and secondary education (PR=1.41; 95% CI: 1.07-1.84), which increased the likelihood of preferring self-sampling. In contrast, cohabiting marital status (aPR=0.63; 95% CI: 0.45-0.88), place of origin in the rest of the coast (aPR=0.67; 95% CI: 0.47-0.96) and Catholic religion (aPR=0.83; 95% CI: 0.73-0.94) decreased the likelihood of preferring self-sampling, with these users leaning toward professional-assisted sampling. None of the behavioral factors, such as age at first sexual intercourse, number of sexual partners, use of contraceptive methods, number of children, PAP history, or history of HPV self-sampling, were significant in the crude analysis. The cognitive factor related to knowledge about HPV was associated with the preference for self-sampling, such that users with a higher level of knowledge (≥ 75th percentile) were less likely to prefer self-sampling (aPR=0.88; 95% CI: 0.71-0.95) (Table 3).

**Table 3:** Crude model and adjusted model of factors associated with self-sampling preference for human papillomavirus detection in women aged 30 to 49 years from the *La Rinconada* Healthcare Center.

Factors		Crude model ^a^		Adjusted model ^b^	
		RPc (95%CI)	p-value	RPa (95%CI)	p-value
Age					
	40-49 years	0.89 (0.76-1.03)	0.118	0.87 (0.75-1.02)	0.091
	30-39 years	Reference		Reference	
Education level					
	Higher education	1.32 (1.02-1.71)	0.031	1.28 (1.01-1.64)	0.044
	Secondary school	1.41 (1.07-1.84)	0.013	1.46 (1.11-1.92)	0.006
	No education/primary school	Reference		Reference	
Marital status					
	Cohabitant	0.63 (0.45-0.88)	0.008	0.61 (0.44-0.83)	0.002
	Married	0.98 (0.84-1.14)	0.819	0.96 (0.83-1.11)	0.647
	Single/widow	Reference		Reference	
Place of origin					
	Abroad	0.95 (0.82-1.11)	0.589	0.97 (0.83-1.12)	0.708
	Jungle	0.98 (0.75-1.27)	0.890	0.97 (0.76-1.24)	0.840
	Rest of the coast	0.67 (0.47-0.96)	0.030	0.70 (0.50-0.97)	0.032
	Metropolitan Lima	Reference		Reference	
Religion					
	Catholic	0.83 (0.73-0.94)	0.004	0.84 (0.74-0.96)	0.007
	Not Catholic	Reference		Reference	
Proximity to the HCC					
	31 min or more	0.98 (0.73-1.31)	0.904	-	-
	11 to 30 min	1.07 (0.81-1.43)	0.599		
	1 to 10 min	Reference			
Employment status					
	Housewife	0.99 (0.85-1.15)	0.939	-	-
	Paid work	Reference			
Age at first sexual intercourse					
	>17 years	0.98 (0.85-1.12)	0.803	-	-
	<17 years	Reference			
Number of sexual partners					
	2+	1.00 (0.87-1.15)	0.965	-	-
	0-1	Reference			
Use of contraceptive methods					
	Yes	0.92 (0.81-1.06)	0.255	-	-
	No	Reference			
Number of children					
	3	1.08 (0.76-1.53)	0.644	-	-
	1 to 2	1.12 (0.79-1.58)	0.499	-	-
	None	Reference			
PAP at any time					
	Yes	0.98 (0.85-1.12)	0.785	-	-
	No	Reference			
Self-sampling history for HPV detection					
	Yes	0.99 (0.81-1.21)	0.925	-	-
	No	Reference			
Knowledge about HPV					
	Percentile ≥ 75	0.85 (0.74-0.97)	0.021	0.88 (0.71-0.95)	0.009
	Percentile<75	Reference		Reference	

a Simple Poisson regression with robust variance.

b Multiple Poisson regression with robust variance.

RPc: crude prevalence ratio, RPa: adjusted prevalence ratio, 95%CI: 95% confidence interval.

HCC: healthcare center, PAP: Pap test, HPV: human papillomavirus.

The adjusted model, which included variables with a p-value <0.20, confirmed that the sociodemographic factors of higher education (RPa=1.28; 95% CI: 1.01-1.64) and secondary education (RPa=1.46; 95% CI: 1.11-1.92) continued to be positively associated with a preference for self-sampling. In contrast, cohabiting marital status (RPa=0.61; 95% CI: 0.44-0.83), being from the rest of the coast (RPa=0.70; 95% CI: 0.50-0.97) and Catholic religion (RPa=0.84; 95% CI: 0.74-0.96) were negatively associated with a preference for self-sampling. In addition, greater knowledge about papillomavirus (RPa=0.88; 95% CI: 0.71-0.95) was inversely associated with a preference for self-sampling (Table 3).

## DISCUSSION

This study revealed that more than three-quarters of users prefered self-sampling for HPV detection (75.6%), which is an encouraging finding, as this strategy could significantly contribute to reducing gaps in cervical cancer prevention. In addition, several sociodemographic factors, such as educational level, marital status, place of origin, and religion, were identified as being associated with a preference for this sampling method. Similarly, greater knowledge about HPV was linked to a lower preference for self-sampling in cervical screening. The magnitude and direction of these associations will be analyzed in detail in the following sections.

This research is one of the first nationwide studies to explore self-sampling preference for HPV detection. The frequency of self-sampling preference for HPV detection was 75.6% (208 users); this finding coincides with the high prevalence reported in studies such as those by Oneko *et al*. ^14^^)^ and Chaw *et al*. ^15^, in which more than half of the users also preferred this method of sample collection (70.0% and 55.7%, respectively). Although we found that the frequency of self-sampling preference was high, a significant percentage preferred sampling assisted by a health professional; this denotes a lack of empowerment and a low predisposition to active cervical cancer prevention, which could be explained by sociodemographic, behavioral, cognitive, and cultural factors, among others reported in previous studies ^4^^,^^16^.

The level of secondary and higher education was significantly associated with a preference for self-sampling; thus, users with higher levels of education were more likely to prefer to obtain the sample themselves to detect HPV. This coincides with the study by Sormani *et al*. ^17^^)^ and Besó *et al*. ^18^, where women with higher levels of education were also more likely to prefer self-sampling because of the security and confidence they had in themselves. In the adjusted analysis, the effect of higher education decreased but remained significant (p=0.044), possibly due to the adjustment for socioeconomic and cognitive variables. Complementarily, women with lower levels of education are more likely to reject self-sampling; therefore, intervening in these sectors of the population with educational sessions on screening options could influence future decisions regarding self-sampling preference, thereby increasing their confidence and preference for self-management of HPV screening.

Cohabitation status was associated with a preference for self-administration; however, due to the absence of previous studies that have explored this factor, it was not possible to establish comparisons, and further research is therefore needed. Similarly, the place of origin “rest of the coast” was associated with a lower probability of preferring self-administration. Ma’som *et al*. ^19^^)^ indicated that the place of origin of participants influences this preference. This aspect should be considered in the implementation of health policies in the many communities of Peru, as each has different beliefs, customs, and previous experiences that may influence the acceptance of self-sampling.

Catholic religion showed a significant association with the preference for self-sampling; however, people who identified with this religion were less likely to prefer it. This finding coincides with the study by Wong *et al*. ^20^, who noted that religion significantly influenced this preference. They observed a fatalistic attitude linked to negative religious coping, whereby health problems were perceived as divine punishment, reducing the willingness to opt for self-sampling in some religious groups. This result contrasts with the study by Ma'som *et al*. ^19^, which found no significant influence of religion on self-sampling preference. In this context, problems related to diagnosis should be addressed through counseling or educational sessions that provide information on the different screening methods available.

Another important finding was that women with higher knowledge of HPV (> 75th percentile) were less likely to prefer self-sampling for human papillomavirus detection. This result differs from that reported by researchers such as Oneko *et al*. ^14^, who found that women with greater knowledge about cervical cancer and HPV were more likely to prefer self-sampling, as in the studies by Adegboyega *et al*. ^21^, Besó *et al*. ^18^, and Gonzales *et al*. ^22^. This discrepancy could be explained by the cultural and contextual conditions of the environment in which the study was conducted. At the primary care level and in predominantly low socioeconomic populations, greater knowledge does not always translate into greater self-efficacy in performing self-administered procedures. Even if women are informed, they may perceive that self-sampling requires technical skills that they prefer to delegate to health personnel, whom they consider more skilled and trustworthy. In addition, sociocultural factors, such as trust in the authority of health personnel and traditional norms, could reinforce the preference for screening by professionals. These findings underscore the importance of designing educational interventions that, in addition to providing information, foster confidence in self-sampling and strengthen perceptions of self-efficacy, especially in vulnerable settings within primary care.

The WHO recommends self-sampling for HPV detection as a complementary method in cervical cancer screening, as well as HPV DNA testing, as it is considered an effective approach for the early detection of this neoplasm in women aged 30 years or older ^1^^,^^23^. Preference for this test could increase participation in cervical cancer prevention programs and, consequently, improve screening coverage. However, this method may not be favorable for women with lower education levels, limited knowledge on the subject, or those from certain religious groups, who may find it more difficult to opt for self-sampling. For this reason, it is recommended to implement educational strategies targeting the different sociocultural profiles of the country.

User preferences regarding the type of device, method, and setting for self-sampling can serve as a basis for developing new or expanded interventions to increase HPV detection ^23^. Studies conducted in several regions of the world, such as Asia, indicate that most participants consider vaginal self-sampling to be easy, convenient, non-embarrassing, comfortable, and reliable ^24^. This has also been reported in low-income countries, such as those in Africa ^25^, whose situation is comparable to the national reality.

Our findings offer a roadmap for health professionals and authorities, suggesting the implementation of differentiated, culturally adapted educational strategies targeting specific groups (women with low levels of education, cohabitants, migrants from other regions, and those with strong religious beliefs) in order to strengthen acceptance of self-sampling as a screening method. In addition, campaigns should be reinforced, focusing not only on knowledge about HPV, but also on increasing confidence in the effectiveness, safety, and ease of self-sampling, especially in the context of primary care and vulnerable populations.

One of the limitations of our study was that the sample was calculated using a formula designed to estimate a proportion, which ensured adequate precision for determining the prevalence of preference for self-sampling for HPV. However, this strategy may not have had sufficient statistical power to evaluate associations between variables, limiting the ability to detect significant relationships. Although an orderly selection of study units was sought, recruiting the same number of cases daily until the sample was complete, it was not completely random (convenience sampling), which represents a limitation that could affect the external validity of our results. In addition, the study was conducted at a single healthcare center, which could reflect particular characteristics of the selected population and not necessarily be applicable to other populations with different sociodemographic, cultural, or healthcare access conditions.

In conclusion, the frequency of preference for self-sampling for HPV detection was high. Likewise, secondary and higher education levels significantly increased the likelihood of preferring this sampling method. On the other hand, marital status, being from coastal regions other than the capital, Catholic religion, and belonging to the highest quartile of knowledge about HPV were negatively associated with the preference for self-sampling, which reduced the likelihood of choosing this screening method.
