# Supplementary material for: Frequency and factors associated with the preference for self-testing for human papillomavirus detection
Source: Rev Peru Med Exp Salud Publica. 2025 Jun 19;42(2):166–74. doi: 10.17843/rpmesp.2025.422.14372 (PMC12377880; doi:10.17843/rpmesp.2025.422.14372)
Supplement: Supplementary material. — Available in the electronic version of the RPMESP. [file rpmesp-42-02-14372-s001.docx]

**Material suplementario 1**

**Instrumento de recolección de datos**

Estimada usuaria, este cuestionario tiene como objetivo determinar la prevalencia y factores asociados a la autotoma para detectar el virus del papiloma humano. A continuación, lea detenidamente cada enunciado y marque con un aspa (X) la respuesta con la que más se identifique. No existen respuestas correctas o incorrectas, estas serán confidenciales y serán utilizadas exclusivamente para fines de investigación.

**a. Preferencia de autotoma para la detección del virus de papiloma humano**

1. En tu próximo tamizaje para la prevención de cáncer de cuello uterino preferirías:

( ) Autotoma

( ) Prueba realizada por el profesional de la salud (Papanicolaou, IVAA, etc.)

**b. Factores sociodemográficos**

2. Edad ( años cumplidos)

3. Máximo grado de instrucción alcanzado

( ) Primaria

( ) Secundaria

( ) Superior

( ) Sin estudios

4. Estado civil

( ) Soltera

( ) Casada

( ) Conviviente

( ) Otro

5. Lugar de origen

( ) Lima metropolitana

( ) Resto de la costa

( ) Sierra

( ) Selva

( ) Extranjero

6. Religión

( ) Católica

( ) Evangelista

( ) Testigo de Jehová

( ) Otro

7. Cercanía al establecimiento de salud

( ) 1-10 minutos

( ) 11-30 minutos

( ) 31 minutos a más

8. Situación laboral

( ) Trabajadora a tiempo completo

( ) Trabajadora a medio tiempo

( ) Ama de casa

**c. Factores conductuales**

9. Inicio de relaciones sexuales

( ) Antes de los 17 años

( ) Después de los 17 años

10. Número de parejas sexuales durante su vida:

11. ¿Cuál de los siguientes métodos anticonceptivos utiliza?

( ) Píldoras

( ) Condón masculino

( ) Inyectable mensual

( ) Inyectable trimestral

( ) Dispositivo intrauterino (T de cobre)

( ) Implante subdérmico

( ) Anticoncepción quirúrgica voluntaria (“ligadura de trompas”)

( ) Otro

( ) No utilizó

12. Número de hijos

( ) Ninguno

( ) Uno

( ) Dos

( ) Tres o más

13. Último Papanicolau

( ) Hace menos de dos años

( ) Hace más de dos años

( ) Nunca

13.1. Si tu respuesta anterior fue “Hace más de dos años” o “Nunca”, ¿Cuáles serían las posibles razones? (Puedes marcar más de una opción)

( ) Vergüenza a que me examinen

( ) Miedo al dolor debido a experiencias previas

( ) Miedo a los posibles resultados

( ) Estoy muy ocupada con las labores del hogar

( ) Estoy muy ocupada con mi trabajo

( ) Nunca he oído hablar del papanicolaou

( ) Lo he olvidado

( ) No recibí ninguna invitación

14. ¿Alguna vez te realizaste la autotoma?

( ) Sí

( ) No

15. Si tu respuesta anterior fue sí, ¿Hace cuánto fue?

( ) Hace menos o igual de 5 años

( ) Más de 5 años

**d. Factor conocimiento**

16. Conocimientos sobre el Virus del Papiloma Humano (VPH)

| Conocimientos | | Si | No | No sé |
| --- | --- | --- | --- | --- |
| a. | El Virus del Papiloma Humano (VPH) puede causar cáncer de cuello uterino |  |  |  |
| b. | Una persona puede tener VPH durante varios años sin saberlo |  |  |  |
| c. | Tener muchas parejas sexuales aumenta el riesgo de contraer el VPH |  |  |  |
| d. | Es muy raro tener VPH |  |  |  |
| e. | El VPH se puede transmitir durante las relaciones sexuales |  |  |  |
| f. | El VPH siempre tiene signos y síntomas visibles |  |  |  |
| g. | El uso de condones reduce el riesgo de contraer el VPH |  |  |  |
| h. | El VPH puede causar VIH/SIDA |  |  |  |
| l. | El VPH puede transmitirse por contacto genital piel a piel |  |  |  |
| j. | Los hombres no pueden contraer el VPH |  |  |  |
| k. | Tener relaciones sexuales a temprana edad aumenta el riesgo de contraer el VPH |  |  |  |
| l. | Hay muchos tipos de VPH |  |  |  |
| m. | El VPH puede causar verrugas genitales |  |  |  |
| n. | El VPH se puede curar con antibióticos |  |  |  |
| o. | La mayoría de las personas sexualmente activas contraerá el VPH en algún momento de su vida |  |  |  |
| p. | El VPH generalmente no necesita ningún tratamiento |  |  |  |
