# Supplementary material for: Frequency and factors associated with the preference for self-testing for human papillomavirus detection
Source: Rev Peru Med Exp Salud Publica. 2025 Jun 19;42(2):166–74. doi: 10.17843/rpmesp.2025.422.14372 (PMC12377880; doi:10.17843/rpmesp.2025.422.14372)
Supplement: Translation

# Translation: Frecuencia y factores asociados a la preferencia de autotoma para detección del virus del papiloma humano

**Authors:** María A. Rosas-Mendoza, Yuly R. Santos-Rosales, Marco A. Chilipio-Chiclla

**Affiliations:** 1 Universidad Nacional Mayor de San Marcos, Facultad de Medicina, Lima, Perú.; 2 Hospital San Juan de Lurigancho, Lima, Perú.

**Keywords:** Pruebas de ADN del Papillomavirus Humano, Virus del Papiloma Humano, Tamizaje Masivo, Autoadministración

## Abstract

**Objetivo.:**

Analizar la frecuencia y los factores asociados a la preferencia de la autotoma para detectar el virus del papiloma humano (VPH) en peruanas de un establecimiento de salud, 2023-2024.

**Materiales y métodos.:**

Se realizo un estudio transversal analítico. La muestra se conformó por 275 mujeres de 30 a 49 años atendidas en el Puesto de Salud Rinconada. La variable principal fue evaluada directamente mediante una pregunta dicotómica, que evidenció la preferencia de la usuaria por esta modalidad de adquisición de muestra cervicouterina para la detección del VPH. El instrumento fue un cuestionario valido y confiable. Se uso estadística multivariada para hallar los factores asociados.

**Resultados.:**

El 75,6% de las usuarias prefiere la autotoma para detectar el VPH. En el análisis ajustado, el grado de instrucción superior (RPa=1,28; IC95%: 1,01-1,64) y secundaria (RPa=1,46; IC95%: 1,11-1,92) incrementaron la probabilidad de preferir la autotoma; mientras que el estado civil conviviente (RPa=0,61; IC95%: 0,44-0,83), la procedencia del resto de la costa (RPa=0,70; IC95%: 0,50-0,97), la religión católica (RPa=0,84; IC95%: 0,74-0,96) y poseer un cuartil superior de conocimientos sobre el VPH (RPa=0,88; IC95%: 0,71-0,95) redujo la probabilidad de preferir la autotoma.

**Conclusión.:**

La frecuencia de la preferencia por la autotoma para detección del VPH es elevada y se asocia con el grado de instrucción secundaria/superior, ser conviviente, proceder de la costa exceptuando la capital, ser católica y tener mayor nivel de conocimientos sobre el VPH.

## INTRODUCCIÓN

El cáncer de cuello uterino, provocado por el virus del papiloma humano (VPH), es un problema de salud pública mundial con altas tasas de mortalidad entre las mujeres, especialmente en países de ingresos bajos ^1^. En el 2022, el Global Cancer Observatory (GLOBOCAN) registró en Perú 4809 nuevos casos de cáncer cervical y 2545 muertes atribuidas a esta causa ^2^.

Esta neoplasia cervical es fácilmente prevenible, y las intervenciones en salud pública pueden ser efectivas para abordar este problema. Entre dichas intervenciones se incluyen los tamizajes como medidas preventivas. En el Perú, se utilizan diferentes tipos de tamizaje, como la citología cervical o prueba de Papanicolaou (PAP), la inspección visual con ácido acético (IVAA) y las pruebas moleculares (autotoma) para la detección del VPH; esta última puede ser recolectada por la propia mujer ^3^. El PAP y la IVAA tienen varios obstáculos que limitan su efectividad, incluidos problemas en la calidad de las muestras, así como barreras socioculturales y psicológicas ^(^^4^^,^^5^. Se ha demostrado que la autotoma es una alternativa aceptable, preferida y eficaz, especialmente en poblaciones vulnerables ^6^^,^^7^, entendiéndose por este término el procedimiento autoadministrado que permite a la usuaria recolectar la muestra cervicovaginal utilizando dispositivos específicos, siguiendo instrucciones estandarizadas y sin la intervención directa de un profesional de la salud ^8^.

La Organización Mundial de la Salud (OMS) recomienda a la autotoma como prueba de tamizaje primario ya que presenta mayor sensibilidad que la citología convencional ^1^, y ha demostrado ser altamente aceptada y preferida en distintas poblaciones de Asia, África y Latinoamérica. En México aumentó la cobertura de tamizaje en poblaciones marginadas, esta experiencia se repitió en Argentina. Además, países como Chile y Colombia han implementado la autotoma como parte de los tamizajes para la prevención de cáncer de cuello uterino ^1^^,^^6^^,^^7^.

La autotoma permite disminuir las brechas existentes en el acceso a las pruebas de tamizaje para el cáncer de cuello uterino, lo que incrementa la cobertura de mujeres tamizadas ^9^. Es crucial evaluar la preferencia hacia la autotoma, ya que esto ofrece un panorama más amplio sobre cómo intervenir de manera efectiva en la población. La disponibilidad de un servicio no garantiza su aceptación o preferencia por parte de la población objetivo. Por lo tanto, comprender este aspecto facilitará la adaptación de las estrategias de salud pública y los programas de prevención para satisfacer las necesidades comunitarias. Esto, a su vez, promoverá una mayor participación, contribuyendo significativamente a la prevención y control del cáncer de cuello uterino.

El puesto de salud La Rinconada es un establecimiento de salud nivel I-2 de atención primaria que brinda servicios preventivos y promocionales, incluyendo el cribado cérvicouterino. En el marco de las estrategias de prevención del cáncer de cérvix promovidas por el Ministerio de Salud (MINSA) viene implementándose el tamizaje del VPH asistido por el profesional de salud. No obstante, con el propósito de ampliar la cobertura y facilitar el acceso a más mujeres, es fundamental conocer sus preferencias respecto a la modalidad de toma de muestra y sus factores asociados. Por lo tanto, el objetivo de este estudio es analizar la frecuencia y los factores asociados a la preferencia de la autotoma para detectar el virus del papiloma humano en peruanas de un puesto de salud durante febrero del 2023 a febrero del 2024.

**MENSAJE CLAVE**

Motivación. El tamizaje cervical es clave para detectar precozmente el cáncer, pero su aceptabilidad limita su realización. Es esencial conocer la preferencia por la autotoma para facilitar su implementación.

Hallazgos. Factores como el nivel de instrucción, estado civil, lugar de origen, religión y conocimiento están asociados con la preferencia de autotoma para detectar el virus del papiloma humano.

Implicancias. Las autoridades sanitarias pueden usar estos hallazgos para fortalecer la promoción de la autotoma a través de campañas educativas, enfocadas en poblaciones con factores que limitan su aceptación, mejorando la cobertura del tamizaje.

## MATERIALES Y MÉTODOS

### Diseño y tipo de investigación

Estudio observacional, analítico y transversal.

### Población y muestra

El estudio se llevó a cabo en el Puesto de Salud La Rinconada, ubicado en San Juan de Miraflores (Lima, Perú). Este establecimiento fue seleccionado por el acceso inmediato que tuvo la investigadora principal. Se trata de un centro de salud de nivel I-2 que atiende a una población de condición socioeconómica predominantemente baja. La recolección de datos se realizó entre diciembre de 2023 y febrero de 2024.

La población comprendió 951 mujeres de 30 a 49 años, cantidad que fue obtenida a partir del padrón de la población asignada al establecimiento. Se incluyeron a las mujeres que aceptaron participar voluntariamente. Se tuvo la previsión de excluir a mujeres con alguna discapacidad; no obstante, durante recolección de datos ninguna presentó discapacidad física, motora o intelectual que impida su participación en el estudio.

Para el cálculo de la muestra se utilizó el software Netquest (https://www.netquest.com/es/panel/calculadora-muestras/calculadoras-estadisticas), considerando un nivel de confianza de 95% y un margen de error de 5%. Para garantizar un diseño estadístico robusto y conservador, se asumió una probabilidad de éxito del 50%, lo que maximiza la varianza de la proporción esperada y asegura el tamaño muestral más grande necesario para alcanzar el nivel de confianza y precisión establecidos. Así, se estimó una muestra de 275 mujeres, seleccionadas de manera no probabilística por conveniencia.

### Variables e instrumento

La variable principal fue la modalidad de toma de muestra para la detección del VPH, evaluada de manera directa a través de la pregunta: “En tu próximo tamizaje para la prevención del cáncer de cuello uterino, ¿qué preferirías?”. Se ofrecieron dos opciones de respuesta: i) “autotoma”, que refleja la preferencia de la usuaria por esta modalidad de recolección de muestra cervicouterina, y ii) “prueba realizada por el profesional de la salud”, que indica la preferencia por la toma asistida por un profesional capacitado. Además, esta forma de medición de la preferencia por la autotoma es consistente con la metodología utilizada en estudios previos ^10^^,^^11^.

Se incluyeron factores sociodemográficos como la edad (30-39/40-49 años), grado de instrucción (sin estudios-primaria/secundaria/superior), estado civil (soltera-viuda/casada/conviviente), lugar de origen (Lima metropolitana/resto de la costa/selva/extranjero), religión (católica/no católica) cercanía al establecimiento de salud (1-10/11-30/31 minutos a más) y situación laboral (trabajo a tiempo completo/trabajo a medio tiempo/ama de casa).

Factores conductuales como el inicio de relaciones sexuales (≤17/>17 años), número de parejas sexuales (cantidad), uso de métodos anticonceptivos (si/no), número de hijos (cantidad), último papanicolaou (menos de dos años/más de dos años/nunca), e historial de realización de la autotoma (si/no).

Factores cognitivos como el conocimiento sobre el virus del papiloma humano (≥percentil75/<percentil75), que se evaluó mediante un cuestionario de 16 ítems dicotómicos (sí/no) que abordaron aspectos relacionados con la transmisión, manifestaciones, epidemiología y manejo del VPH, el cual fue adaptado del estudio publicado de Marlow *et al*. ^12^. Se asignó un punto por cada respuesta correcta y se estableció un punto de corte en el percentil 75 para identificar a las mujeres que se encontraban en el cuartil superior de conocimientos.

El instrumento fue un cuestionario (material suplementario 1) validado por juicio de cinco expertos. Dos de ellos eran profesionales obstetras con experiencia metodológica, y tres contaban con amplio conocimiento en la prevención del cáncer ginecológico. La validez de contenido se evaluó mediante el coeficiente V de Aiken, obteniéndose un valor de V=0,995, lo que indica una alta validez. Para determinar la confiabilidad, se utilizó el coeficiente alfa de Cronbach; para ello, se aplicó una prueba piloto a 30 mujeres en edad fértil del mismo establecimiento, no incluidas en la muestra final. El cálculo del coeficiente se realizó solo con ítems de respuesta politómica. Como resultado se obtuvo que el cuestionario tiene una confiabilidad alta de 0,72, lo cual verificó su fiabilidad.

### Procedimientos

Tras obtener los permisos correspondientes, se aplicaron las encuestas en el Puesto de Salud La Rinconada. Durante los 75 días hábiles del período de estudio (diciembre de 2023 a febrero de 2024), se seleccionaron diariamente cuatro usuarias a partir de las citas programadas. Solo se incluyó a aquellas que aceptaron participar y firmaron el consentimiento informado. Las encuestas fueron autoadministradas en formato impreso y se realizaron en la sala de espera de los consultorios de obstetricia, siendo completadas por las propias usuarias de manera individual.

### Plan de análisis

Los datos fueron almacenados en una base de Excel, donde se eliminó todos aquellos registros de participantes que presentaron ausencia de información o inconsistencias. El procesamiento de datos se realizó en el Software Statistical Package for the Social Sciences (SPSS) versión 26. Para el análisis descriptivo de la variable cuantitativa edad, se utilizó la mediana como medida de tendencia central y el rango intercuartílico (RIC) como medida de dispersión, tras verificar la no normalidad de los datos mediante la prueba de Kolmogorov-Smirnov. En el análisis descriptivo de variables cualitativas, se emplearon las frecuencias absolutas (n) y relativas (%). En el análisis bivariado, se utilizó la prueba Chi cuadrado de Pearson con un nivel de confianza del 95%. Finalmente, se estimaron las razones de prevalencia cruda (RPc) y ajustada (RPa) mediante regresión de Poisson con varianza robusta, utilizando la interfaz de Modelos Lineales Generalizados. En el modelo ajustado, solo se incluyeron variables con un valor de p<0,20 en el modelo crudo, siguiendo el criterio de Hosmer y Lemeshow ^13^, para reducir el riesgo de omitir variables relevantes y considerar posibles factores de confusión.

### Aspectos éticos

Se respetó los criterios de Helsinki y bioéticos en investigación humana. El proyecto fue revisado y aprobado por el comité de ética de la Facultad de Medicina de la Universidad Nacional Mayor de San Marcos (Acta N°0152-2023) y el comité de ética de la Dirección de Redes Integradas de Salud (DIRIS) Lima Sur (N° 047-2023).

## RESULTADOS

Las características generales de las 275 mujeres mostraron una edad mediana de 37,0 años (RIC: 33,0-43,0). La mayoría perteneció al grupo etario de 30 a 39 años (62,2%), tenía un grado de instrucción superior (60,4%) y estaba casada (66,9%). La mayoría provenía de Lima metropolitana (56,0%), seguida por aquellas de origen extranjero (27,3%). Además, la mayoría profesaba la religión católica (65,8%), tenía una cercanía al establecimiento de salud de 31 minutos o más (48,7%) y se encontraba en situación laboral de ama de casa (71,3%) (tabla 1).

**Tabla 1:** Características generales de las mujeres de 30 a 49 años atendidas en consultorio de obstetricia del Puesto de Salud La Rinconada.

Características generales		n	%
Edad^a^		37,0 (33,0-43,0)	
	30-39 años	171	62,2
	40-49 años	104	37,8
Grado de instrucción			
	Superior	166	60,4
	Secundaria	63	22,9
	Primaria	3	1,1
	Sin estudios	43	15,6
Estado civil			
	Casada	184	66,9
	Soltera	44	16,0
	Conviviente	39	14,2
	Viuda	8	2,9
Lugar de origen			
	Lima metropolitana	154	56,0
	Resto de la costa	28	10,2
	Selva	18	6,5
	Extranjero	75	27,3
Religión			
	Católica	181	65,8
	Evangelista	15	5,5
	Testigo de Jehová	3	1,1
	Otros^b^	76	27,6
Cercanía al establecimiento de salud			
	31 min a más	134	48,7
	11 a 30 min	122	44,4
	1 a 10 min	19	6,9
Situación laboral			
	Trabajo a tiempo completo	29	10,5
	Trabajo a medio tiempo	50	18,2
	Ama de casa	196	71,3
Total		275	100,0

a Mediana y rango intercuartílico, variable cuantitativa sin distribución normal (Prueba de Kolmogorov Smirnov p<0,001).

b Incluye a quienes manifestaron no profesar ninguna religión.

El 75,6% (208) de las mujeres evaluadas reportaron preferencia por la autotoma para la detección del VPH, mientras que el 24,4% (67) mostró preferencia por la toma asistida por un profesional de la salud.

El análisis bivariado reveló diferencias significativas entre las mujeres que preferían la autotoma y aquellas que no, en cuanto a grado de instrucción (p=0,010), estado civil (p=0,001), lugar de origen (p=0,036), religión (p=0,008) y nivel de conocimiento sobre el VPH (p=0,019). Las mujeres que preferían la autotoma generalmente tenían un mayor nivel de instrucción (superior: 62,0%; secundaria: 25,0%). Por el contrario, las que mostraron preferencia por la autotoma fueron en menor frecuencia convivientes (9,6%), católicas (61,5%) y con un conocimiento sobre VPH ≥ percentil 75 (44,7%). Otras variables como la edad, cercanía al establecimiento de salud, situación laboral, edad de inicio de relaciones sexuales, número de parejas sexuales, uso de métodos anticonceptivos, número de hijos, historial de PAP previo y antecedentes de autotoma VPH no mostraron diferencias significativas (p>0,05) según la preferencia por la autotoma (tabla 2).

**Tabla 2:** Análisis bivariado de las características sociodemográficas, conductuales y de conocimiento según preferencia de la autotoma para la detección del VPH en mujeres de 30 a 49 años en el Puesto de Salud La Rinconada.

Características		Sí prefiere la autotoma	No prefiere la autotoma	Valor de p^a^
		n (%)	n (%)	
Edad				
	40-49 años	73 (35,1)	31 (46,3)	0,101
	30-39 años	135 (64,9)	36 (53,7)	
Grado de instrucción				
	Superior	129 (62,0)	37 (55,2)	0,010
	Secundaria	52 (25,0)	11 (16,4)	
	Sin estudios/primaria	27 (13,0)	19 (28,4)	
Estado civil				
	Conviviente	20 (9,6)	19 (28,4)	0,001
	Casada	146 (70,2)	38 (56,7)	
	Soltera/viuda	42 (20,2)	10 (14,9)	
Lugar de origen				
	Resto de la costa	15 (7,2)	13 (19,3)	0,036
	Selva	14 (6,7)	4 (6,0)	
	Extranjero	57 (27,4)	18 (26,9)	
	Lima metropolitana	122 (58,7)	32 (47,8)	
Religión				
	Católica	128 (61,5)	53 (79,1)	0,008
	No católica^b^	80 (38,5)	14 (20,9)	
Cercanía al establecimiento de salud				
	31 min a más	97 (46,6)	37 (55,2)	0,407
	11 a 30 min	97 (46,6)	25 (37,3)	
	1 a 10 min	14 (6,8)	5 (7,5)	
Situación laboral				
	Ama de casa	148 (71,2)	48 (71,6)	0,939
	Trabajo remunerado	60 (28,8)	19 (28,4)	
Edad de inicio de relaciones sexuales				
	>17 años	130 (62,5)	43 (64,2)	0,805
	< 17 años	78 (37,5)	24 (35,8)	
Número de parejas sexuales				
	2 +	131 (63,0)	42 (62,7)	0,965
	0-1	77 (37,0)	25 (37,3)	
Uso de métodos anticonceptivos				
	Sí	95 (45,7)	36 (53,7)	0,251
	No	113 (54,3)	31 (46,3)	
Número de hijos				
	3	91 (43,7)	31 (46,3)	0,702
	1 a 2	106 (51,0)	31 (46,3)	
	Ninguno	11 (5,3)	5 (7,4)	
PAP alguna vez				
	Sí	83 (39,9)	28 (41,8)	0,784
	No	125 (60,1)	39 (58,2)	
Antecedente de autotoma para detectar el VPH				
	Sí	27 (13,0)	9 (13,4)	0,924
	No	181 (87,0)	58 (86,6)	
Conocimientos sobre el VPH				
	Percentil>75	93 (44,7)	41 (61,2)	0,019
	Percentil<75	115 (53,3)	26 (38,8)	
Total		208 (100,0)	67 (100,0)	

a Prueba de Chi cuadrado de Pearson.

b Incluye a quienes manifiestan no profesar ninguna religión.

PAP: prueba de Papanicolaou, VPH: virus del papiloma humano.

En el análisis crudo, los factores sociodemográficos asociados con la preferencia por la autotoma fueron el grado de instrucción superior (RPc=1,32; IC95%: 1,02-1,71) y secundario (RPc=1,41; IC95%: 1,07-1,84), lo que aumentó la probabilidad de preferir la autotoma. En cambio, el estado civil conviviente (RPc=0,63; IC95%: 0,45-0,88), el lugar de origen en el resto de la costa (RPc=0,67; IC95%: 0,47-0,96) y la religión católica (RPc=0,83; IC95%: 0,73-0,94) disminuyeron las probabilidades de preferir la autotoma, inclinándose estas usuarias por la toma asistida por un profesional. Ninguno de los factores conductuales, como la edad de inicio de relaciones sexuales, número de parejas sexuales, uso de métodos anticonceptivos, número de hijos, historial de PAP o antecedentes de autotoma VPH, resultaron significativos en el análisis crudo. El factor cognitivo relacionado con el conocimiento sobre VPH sí se asoció con la preferencia por la autotoma, de modo que las usuarias con un nivel de conocimiento mayor (≥ percentil 75) tuvieron menores probabilidades de preferir la autotoma (RPc=0,88; IC95%: 0,71-0,95) (tabla 3).

**Tabla 3:** Modelo crudo y modelo ajustado de factores asociados a la preferencia de autotoma para detección del virus del papiloma humano en mujeres de 30 a 49 años en el Puesto de Salud La Rinconada.

Factores		Modelo crudo^a^		Modelo ajustado^b^	
		RPc (IC95%)	Valor de p	RPa (IC95%)	Valor de p
Edad					
	40-49 años	0,89 (0,76-1,03)	0,118	0,87 (0,75-1,02)	0,091
	30-39 años	Referencia		Referencia	
Grado de instrucción					
	Superior	1,32 (1,02-1,71)	0,031	1,28 (1,01-1,64)	0,044
	Secundaria	1,41 (1,07-1,84)	0,013	1,46 (1,11-1,92)	0,006
	Sin estudios/primaria	Referencia		Referencia	
Estado civil					
	Conviviente	0,63 (0,45-0,88)	0,008	0,61 (0,44-0,83)	0,002
	Casada	0,98 (0,84-1,14)	0,819	0,96 (0,83-1,11)	0,647
	Soltera/viuda	Referencia		Referencia	
Lugar de origen					
	Extranjero	0,95 (0,82-1,11)	0,589	0,97 (0,83-1,12)	0,708
	Selva	0,98 (0,75-1,27)	0,890	0,97 (0,76-1,24)	0,840
	Resto de la costa	0,67 (0,47-0,96)	0,030	0,70 (0,50-0,97)	0,032
	Lima metropolitana	Referencia		Referencia	
Religión					
	Católica	0,83 (0,73-0,94)	0,004	0,84 (0,74-0,96)	0,007
	No católica	Referencia		Referencia	
Cercanía al EESS					
	31 min a más	0,98 (0,73-1,31)	0,904	-	-
	11 a 30 min	1,07 (0,81-1,43)	0,599		
	1 a 10 min	Referencia			
Situación laboral					
	Ama de casa	0,99 (0,85-1,15)	0,939	-	-
	Trabajo remunerado	Referencia			
Edad de inicio de relaciones sexuales					
	>17 años	0,98 (0,85-1,12)	0,803	-	-
	<17 años	Referencia			
Número parejas sexuales					
	2+	1,00 (0,87-1,15)	0,965	-	-
	0-1	Referencia			
Uso de métodos anticonceptivos					
	Sí	0,92 (0,81-1,06)	0,255	-	-
	No	Referencia			
Número de hijos					
	3	1,08 (0,76-1,53)	0,644	-	-
	1 a 2	1,12 (0,79-1,58)	0,499	-	-
	Ninguno	Referencia			
PAP alguna vez					
	Sí	0,98 (0,85-1,12)	0,785	-	-
	No	Referencia			
Antecedente autotoma para detectar el VPH					
	Sí	0,99 (0,81-1,21)	0,925	-	-
	No	Referencia			
Conocimientos sobre el VPH					
	Percentil ≥ 75	0,85 (0,74-0,97)	0,021	0,88 (0,71-0,95)	0,009
	Percentil<75	Referencia		Referencia	

a Regresión de Poisson simple con varianza robusta.

b Regresión de Poisson múltiple con varianza robusta.

RPc: razón de prevalencia cruda, RPa: razón de prevalencia ajustada, IC95%: intervalo de confianza al 95%.

EESS: establecimiento de salud, PAP: prueba de Papanicolaou, VPH: virus del papiloma humano.

El modelo ajustado, que incluyó variables con valor p<0,20, corroboró que los factores sociodemográficos grado de instrucción superior (RPa=1,28; IC95%: 1,01-1,64) y secundario (RPa=1,46; IC95%: 1,11-1,92) seguían asociándose positivamente con la preferencia por la autotoma. En cambio, el estado civil conviviente (RPa=0,61; IC95%: 0,44-0,83), la procedencia del resto de la costa (RPa=0,70; IC95%: 0,50-0,97) y la religión católica (RPa=0,84; IC95%: 0,74-0,96) se asociaron negativamente con la preferencia por la autotoma. Además, un mayor conocimiento sobre el papilomavirus (RPa=0,88; IC95%: 0,71-0,95) se asoció inversamente con la preferencia por la autotoma (tabla 3).

## DISCUSIÓN

El presente estudio reveló que más de tres cuartas partes de las usuarias prefieren la autotoma de muestras para la detección del VPH (75,6%), lo cual constituye un hallazgo alentador, ya que esta estrategia podría contribuir significativamente a reducir las brechas en la prevención del cáncer de cérvix. Además, se identificaron varios factores sociodemográficos, como el grado de instrucción, estado civil, lugar de origen y religión, asociados con la preferencia por esta modalidad de toma de muestras. De igual forma, el mayor nivel de conocimiento sobre el VPH se vinculó con la menor predilección por la autotoma en el tamizaje cervicouterino. La magnitud y dirección de estas asociaciones se analizarán en detalle en las secciones siguientes.

Esta investigación es uno de los primeros estudios a nivel nacional que explora la preferencia de autotoma de muestra para la detección del VPH. La frecuencia de la preferencia de autotoma para detección del VPH fue 75,6% (208 usuarias); este hallazgo, coincide con la alta prevalencia reportada en estudios como Oneko *et al.*^14^ y Chaw *et al.* ^(^^15^ donde más de la mitad de las usuarias también prefirió esta modalidad de obtención de muestra (70,0% y 55,7%, respectivamente). Si bien, se halló una alta frecuencia de preferencia de autotoma, un porcentaje importante prefirió la toma asistida por un profesional de salud; esto denota la falta de empoderamiento y la escasa predisposición para la prevención activa del cáncer de cérvix, lo cual se explicaría a partir de factores sociodemográficos, conductuales, cognitivos, culturales, entre otros reportados en estudios previos ^4^^,^^16^.

El grado de instrucción secundaria y superior se asociaron significativamente con la preferencia de la autotoma; de tal manera, que las usuarias con mayor escolaridad tuvieron mayor predilección para gestionar ellas mismas la toma de muestra para detectar el VPH, esto coincide con el estudio de Sormani *et al.*^17^ y Besó *et al.*^18^, donde las mujeres con mayor escolaridad también tuvieron más probabilidad de preferir el autotamizaje por la seguridad y confianza que tenían consigo mismas. En el análisis ajustado, el efecto de la educación superior decreció, pero se mantuvo significativo (p=0,044), posiblemente debido al ajuste por variables socioeconómicas y cognitivas. Complementariamente, las mujeres con menor grado de instrucción tienen más probabilidad de rechazar la autotoma; por ello, intervenir en estos sectores de la población con sesiones educativas sobre las opciones de pruebas de tamizaje, podría influir en la decisión a futuro de la preferencia de la autotoma, aumentando de esta manera su confianza y preferencia por la modalidad de autogestión del tamizaje para detectar el VPH.

El estado civil de conviviente se asoció con la preferencia por la autotoma; sin embargo, debido a la ausencia de estudios previos que hayan explorado este factor, no fue posible establecer comparaciones, por lo que se requieren nuevas investigaciones al respecto. Asimismo, el lugar de origen «resto de la costa» se asoció con una menor probabilidad de preferir la autotoma. Ma’som *et al*. ^19^ indicaron que el lugar de origen de las participantes influye en dicha preferencia. Este aspecto debe considerarse en la implementación de políticas de salud en las diversas comunidades del Perú, ya que cada una presenta creencias, costumbres y experiencias previas distintas que pueden influir en la aceptación de la autotoma.

La religión católica mostró una asociación significativa con la preferencia por la autotoma; sin embargo, las personas que se identificaban con esta religión presentaron una menor probabilidad de preferirla. Este hallazgo coincide con el estudio de Wong *et al.*^20^*,* quienes señalaron que la religión influyó significativamente en dicha preferencia. Observaron una actitud fatalista vinculada al afrontamiento religioso negativo, según la cual los problemas de salud eran percibidos como una penitencia divina, lo que reducía la disposición a optar por la autotoma en algunos grupos religiosos. Este resultado contrasta con el estudio de Ma’som *et al*. ^(^^19^^)^ donde no se encontró una influencia significativa de la religión en la preferencia por la autotoma. En este contexto, los problemas relacionados con el diagnóstico deben abordarse mediante consejerías o sesiones educativas que informen sobre los distintos métodos de tamizaje disponibles.

Otro hallazgo importante fue que las mujeres con un cuartil superior de conocimientos sobre el VPH (> percentil 75) tuvieron menor probabilidad de preferir la autotoma del test para la detección del papilomavirus humano. Este resultado difiere de lo reportado por investigadores como Oneko *et al.* ^(^^14^ quienes indican que las mujeres con mayor conocimiento sobre el cáncer de cuello uterino y el VPH tienen más probabilidades de preferir la autotoma, al igual que en los estudios de Adegboyega *et al.*^21^, Besó *et al.* ^(^^18^ y Gonzales *et al.* ^(^^22^. Esta discrepancia podría explicarse por las condiciones culturales y contextuales del entorno en el que se realizó el estudio. En el nivel de atención primaria y en poblaciones predominantemente de bajo nivel socioeconómico, un mayor conocimiento no siempre se traduce en una mayor autoeficacia para realizar procedimientos autoadministrados. Aunque las mujeres estén informadas, pueden percibir que la autotoma requiere habilidades técnicas que prefieren delegar al personal de salud, a quien consideran más capacitado y confiable. Además, factores socioculturales, como la confianza en la autoridad del personal de salud y las normas tradicionales, podrían reforzar esta preferencia por el tamizaje realizado por profesionales. Estos hallazgos subrayan la importancia de diseñar intervenciones educativas que, además de proporcionar información, fomenten la confianza en la autotoma y fortalezcan la percepción de autoeficacia, especialmente en contextos vulnerables dentro de la atención primaria.

La OMS recomienda la autotoma de muestras para la detección del VPH como método complementario en el tamizaje del cáncer de cuello uterino, así como la prueba de ADN del VPH, por considerarse un enfoque eficaz para la detección precoz de esta neoplasia en mujeres de 30 años o más ^(^^1^^,^^23^. La preferencia por esta prueba podría aumentar la participación en los programas de prevención del cáncer de cuello uterino y, en consecuencia, mejorar la cobertura del tamizaje. Sin embargo, este método podría no ser favorable para mujeres con menor nivel de instrucción, escaso conocimiento sobre el tema o pertenecientes a ciertos grupos religiosos, quienes podrían tener mayor dificultad para optar por la autotoma. Por tal motivo, se recomienda implementar estrategias educativas dirigidas a los distintos perfiles socioculturales del país.

Las preferencias de las usuarias en relación con el tipo de dispositivo, el método y el entorno para la autotoma pueden servir de base para desarrollar intervenciones nuevas o ampliadas que incrementen la detección del VPH ^23^. Estudios realizados en diversas regiones del mundo, como Asia, indican que la mayoría de las participantes consideran la autotoma vaginal como una práctica fácil, conveniente, no vergonzosa, cómoda y confiable ^24^. Esto también se ha observado en países de bajos recursos, como en África ^25^, cuya situación es comparable con la realidad nacional.

Los hallazgos ofrecen una hoja de ruta para los profesionales y autoridades en salud, sugiriendo la implementación de estrategias educativas diferenciadas, culturalmente adaptadas y dirigidas a grupos específicos (mujeres con bajo nivel educativo, convivientes, migrantes de otras regiones y aquellas con creencias religiosas marcadas), con el fin de fortalecer la aceptación de la autotoma como método de tamizaje. Además, se recomienda reforzar las campañas, no solo enfocándose en el conocimiento sobre el VPH, sino también en incrementar la confianza en la eficacia, seguridad y facilidad de la autotoma, especialmente en el contexto de atención primaria y poblaciones vulnerables.

Es importante señalar como limitación del presente estudio que la muestra fue calculada utilizando una fórmula diseñada para estimar una proporción, lo que garantizó una adecuada precisión para determinar la prevalencia de la preferencia por la autotoma del VPH. Sin embargo, esta estrategia podría no haber tenido el poder estadístico suficiente para evaluar asociaciones entre variables, limitando la capacidad para detectar relaciones significativas. A pesar de que se procuró una selección ordenada de las unidades de estudio, reclutando la misma cantidad de casos diariamente hasta completar la muestra, esta no fue completamente aleatoria (muestreo por conveniencia), lo que representa una limitación que podría afectar la validez externa del estudio. Además, el estudio se realizó en un solo puesto de salud, lo que podría reflejar características particulares de la población seleccionada y no necesariamente ser aplicables a otras poblaciones con diferentes condiciones sociodemográficas, culturales o de acceso a la salud.

En conclusión, la frecuencia de la preferencia por la autotoma de muestras para la detección del VPH fue alta. Asimismo, los niveles educativos secundario y superior aumentaron significativamente la probabilidad de preferir esta modalidad de toma de muestra. Por otro lado, los factores estado civil de conviviente, procedencia de regiones costeras fuera de la capital, religión católica y pertenecer al cuartil superior de conocimientos sobre el VPH se asociaron negativamente con la preferencia por la autotoma, lo que redujo la probabilidad de optar por esta modalidad de tamizaje.
